# BMC Anesthesiology reviewer acknowledgement, 2013

**DOI:** 10.1186/1471-2253-14-7

**Published:** 2014-02-13

**Authors:** Thomas A Rowles

**Affiliations:** 1 BioMed Central Floor 6, 236 Gray’s Inn Road, London WC1X 8HB, UK

## Abstract

**Contributing reviewers:**

The editors of BMC Anesthesiology would like to thank all our reviewers who have contributed their time to the journal in Volume 13 (2013).

## 

Antonella Agodi

Italy

Terrence Allen

USA

Wilson Alves-Do-Prado

Brazil

Barbara Antuna-Puente

Mexico

Marco Baciarello

Italy

Angela Bader

USA

Sukhminder Bajwa

India

Brian Baldo

Australia

Michael Barrington

Australia

Mary Barry

Ireland

Georgia Beasley

USA

Alex Bekker

USA

Dan Benhamou

France

Daniele Biasucci

Italy

Horst Bickel

Germany

Edward Bittner

USA

Simon Body

USA

Peter Bragge

Australia

Gerhard Brodner

Germany

Donal Buggy

Ireland

John Butterworth

USA

Enrico Camporesi

USA

Xavier Capdevila

France

Annalisa Carlucci

Italy

Michele Carron

Italy

George Carvalho

Canada

Davide Cattano

USA

Vidya Chidambaran

USA

Daniel Chipman

USA

Hovig Chitilian

USA

Eric Chudler

USA

Francois Clergue

Switzerland

Jean-Marie Conil

France

Boris Cruz

Brazil

Philipp Dahm

USA

Abdelazeem Dawlatly

Albania

Alberto De Armendi

USA

Gildasio De Oliveira

USA

Mohamed Bilal Delvi

Saudi Arabia

Anahat Dhillon

USA

Matteo Di Nardo

Italy

Ambra Licia Di Prima

Italy

Dawn Dillman

USA

Prakash Dubey

India

Leopold Eberhart

Germany

Roderic Eckenhoff

USA

Christoph Bernhard Eich

Germany

Gunnar Elke

Germany

Martin Esken

Canada

Miguel Ferrer

Spain

Lee Fleisher

USA

Carlos Flores

Spain

Stuart Forman

USA

Vincent Fraipont

Belgium

Tibor Fulop

USA

Javier Garcia-Campayo

Spain

Allan Garland

Canada

Marco Gemma

Italy

Katja Goetz

Germany

Nermin Gogus

Turkey

Alejandro González-Ojeda

Mexico

Alberto Grassetto

Italy

Robert Greenberg

USA

Burak Guclu

Turkey

Robert G Hahn

Sweden

Christiane Hartog

Germany

Avijit Hazra

India

David Healy

USA

Margareta Hedström

Sweden

Gregg Homanics

USA

Jennifer Hunter

United Kingdom

Andreas Janata

USA

Vesna Jevtovic-Todorovic

USA

Stephanie Jones

USA

Girish Joshi

USA

David Kaczka

USA

Rebecca Kalman

USA

Takashi Kawasaki

Japan

Mohammad Reza Khajavi

Iran

Jeffrey Kirsch

USA

Moritz A. Konerding

Germany

Aaron Kopman

USA

Keith Kowalczyk

USA

Morten Tange Kristensen

Denmark

Giovanni Landoni

Italy

Chiara Lazzeri

Italy

Francois Lellouche

Canada

Hendrikus Lemmens

USA

Harald Lenz

Norway

Erik Lichnovsky

United Kingdom

Myong Cheol Lim

South Korea

Steven Lisco

USA

Carl Lombard

South Africa

Nadia Lunardi

USA

Troels Haxholdt Lunn

Denmark

Carl Lynch

USA

Laxmaiah Manchikanti

USA

Maria Martin-Cancho

Spain

Tadashi Matsuura

Japan

Tommaso Mauri

Italy

Lynne Maxwell

USA

Angela Meier

USA

Pavel Michalek

Czech Republic

Søren Mikkelsen

Denmark

Vincent Minville

France

Constance Monitto

USA

Jane Muret

France

Hiroto Narimatsu

Japan

Lone Nikolajsen

Denmark

Eveline Nueesch

United Kingdom

Yen-Chuan Ou

Taiwan

Mehmet Ozcan

USA

Giovanni Pacini

Italy

Christian Pagnoux

France

Peter Pan

USA

Martyn Parker

United Kingdom

Douglas Paull

USA

Rupert Pearse

United Kingdom

Pernille Petersen

Denmark

Annop Piriyapatsom

USA

Neil Pollock

New Zealand

Valentina Porta

Brazil

Jean-Pierre Quenot

France

Peter Quesenberry

USA

Jacob Raphael

USA

Lars Rasmussen

Denmark

Jochen Renner

Germany

Daniel Reuter

Germany

Sheila Riazi

Canada

Reitze Rodseth

South Africa

Stefano Romagnoli

Italy

Parvin Sajedi

Iran

Michael Sander

Germany

Raffaele Scala

Italy

Dirk Schadler

Germany

Michael Schaefer

Germany

Gereon Schaelte

Germany

Stephan Schug

Australia

Lex Schultheis

USA

Marcus J Schultz

Netherlands

Wolfram Schummer

Germany

Konrad Schwarzkopf

Germany

Karin Sennfält

Sweden

Reza Shariat Moharari

Iran

Martin Soerensen

Denmark

Ju-Tae Sohn

South Korea

Massimiliano Sorbello

Italy

Peter Spieth

Germany

Bernd Stegmayr

Sweden

Bala Subramaniam

USA

Rajeev Subramanyam

USA

Koichi Suehiro

Japan

Richard Szydlo

United Kingdom

Hirotaka Tashiro

Japan

Vladimir Tesar

Czech Republic

Lorenz Theiler

Switzerland

Ronan Thibault

Switzerland

Joseph Tobin

USA

Kamil Toker

Turkey

Mark Tommerdahl

USA

Mei-Yung Tsou

Taiwan

Melek Tulunay

Turkey

Betty Tyler

USA

Mikko Uusi-Oukari

Finland

David Van Duin

USA

Christian Von Heymann

Germany

Dagmar Westerling

Sweden

Chris Winkelman

USA

Hermann Wrigge

Germany

Victor Xia

USA

Zhongcong Xie

USA

Xu Xuzhong

China

Massimo Zambon

Italy

Sebastian Zaremba

USA

Lin Zou

USA

## Pre-publication history

The pre-publication history for this paper can be accessed here:

http://www.biomedcentral.com/1471-2253/14/7/prepub

